# Cancer growth and metastasis as a metaphor of Go gaming: An Ising model approach

**DOI:** 10.1371/journal.pone.0195654

**Published:** 2018-05-02

**Authors:** Didier Barradas-Bautista, Matias Alvarado-Mentado, Mark Agostino, Germinal Cocho

**Affiliations:** 1 ABACUS Project, CINVESTAV, Mexico City, Mexico; 2 Computer Science Department, CINVESTAV, Mexico City, Mexico; 3 Curtin Health Innovation Research Institute and Curtin Institute Computation, Curtin University, Perth, Australia; 4 Complex Sciences Center, UNAM, Mexico City, Mexico; 5 Physics Institute, UNAM, Mexico City, Mexico; University of South Alabama Mitchell Cancer Institute, UNITED STATES

## Abstract

This work aims for modeling and simulating the metastasis of cancer, via the analogy between the cancer process and the board game Go. In the game of Go, black stones that play first could correspond to a metaphor of the birth, growth, and metastasis of cancer. Moreover, playing white stones on the second turn could correspond the inhibition of cancer invasion. Mathematical modeling and algorithmic simulation of Go may therefore benefit the efforts to deploy therapies to surpass cancer illness by providing insight into the cellular growth and expansion over a tissue area. We use the Ising Hamiltonian, that models the energy exchange in interacting particles, for modeling the cancer dynamics. Parameters in the energy function refer the biochemical elements that induce cancer birth, growth, and metastasis; as well as the biochemical immune system process of defense.

## Introduction

The dominant pathology and mortality today is due to diseases such as cancer, diabetes, and cardiovascular events [1]. Common features of these diseases are to have a long latency period and to be related to a large number of causal factors. Therefore, we might call them complex diseases. In modern times, these diseases imply strong social and financial problems and a heavy burden for the health systems [2, 3]. Of these factors, some of them are causal (negative) factors of the disease [4, 5] and others preventive (positive) factors [6, 7]. There is no absolute dominant factor, but all of them have the same “weight”, the diseases complexity [8–11] and the need to adopt novel approaches, particularly for cancer [12–16]. In recent decades, numerous studies have identified common characteristics, so-called “hallmarks” [17], that allow the survival and growth of cells to become cancerous tumors [18–27], and are present across different types of cancer [28–35]. These hallmarks include sustained proliferative signaling [23], evading of growth suppressors [12], replicative immortality [18, 21, 22, 24], increased invasive capacity [36], resistance of cell death [37], induction of angiogenesis [38–40], and ability to undergo metastasis [20, 36, 41–45]. The continued research around cancer expanded these hallmarks to include the deregulation of metabolism, immune system evasion, genomic instability, and tumor-promoted inflammation. Altogether, these characteristics are part of the driving program for cancer cells successfully spread and control its growth and resources.

Go is a two player, zero-sum and complete information game, black versus white stones, played on a board of 19 x 19 grid. A Go gaming state is a configuration given by the combination of black/white/empty board positions [46], http://senseis.xmp.net. The goal of game is to gain the most territory of the board. At each turn, each player places one black/white stone on an empty board cross-point position. Black plays first, and white receives a compensation in the score known as *komi*, by playing the second turn. Same color stones joined in horizontal or vertical lines become one indivisible compound stone. Hence, single or long stones are struggling for achieving territory control. One stone’s *liberty* is any contiguous vacant board cross-point in the vertical or horizontal direction. If a stone has zero liberties because is surrounded by the adversary that stone can be removed from the board, this is referred as a *capture*. An *eye* is a single liberty shared by four same color stones. Placement of a stone in an adversary indestructible eye is a direct capture or *suicide*, which is not allowed. A stone is *alive* while it cannot be captured and *dead* if it cannot avoid being captured. The game ends when both players pass a turn. The score is computed based on both board territory occupied and the number of single adversary stones captured. The winner is declared as the player having the most extensive territorial control and the highest number of captures.

A triumph in a Go match requires complex strategies that use a range of simple tactics [46]. For humans or computer Go players, the hardest task is to assess how to deploy stones to better the control of the board over the adversary player at any game stage. It means to decide the stone allocation for the next play regarding the current board configuration and mind to enforce the synergy of its stones [47, 48]. The analysis and algorithmic development of Go have been in the core advances in computer science this century, and in 2017, a categorical triumph of the AlphaGo machine winning more than 60 simultaneous games against the top Go human player is the definitive triumph of Go game computational intelligence over Go humans competences [49].

In this work we model the conflict between metastatic dynamics and the action of the immunologic system, using as a metaphor this clever game for strategic control of regions.

### Ising model

Ising model describes magnetic properties of materials from the interactions of constituent atomic spins, as elementary magnetic moments which possess a dichotomy behavior that points randomly in the up or down directions, or formal dichotomy value 1 or −1 [50]. Each spin interacts with neighboring spins or with external fields that tend to align them in the applied direction, and depending on the strength of interactions, the whole system gets phase transitions among new spin clusters domains, percolate through the entire system, or fill out whole regions of the material [51]. The spins are arranged in an N-dimensional lattice, the interaction energy of which is described by the Hamiltonian:

$$\begin{array}{c} H=\sum_{ij}w_{ij}x_{i}x_{j}-\mu \sum_{i}h_{i}x_{i} \end{array}$$ (1)

*w*_*ij*_ sets for interaction between spin *i* and *j*, *μ* is the magnitude of an external magnetic field, and *h*_*i*_ the magnetic field contribution at site *i*; for a homogeneous external field, *h*_*i*_ = 1. The Ising model as the basis for the modeling, algorithmic setting and simulation of stochastic behave, describes the interaction of the magnetic field in two materials, allowing to observe the phase transition as the sudden changes in the energy where the materials change their state.

In the struggling for board area control in Go, the Ising model is relevant to modelling the dynamics of complex interaction, henceforth for designing algorithms to quantify the synergy among allied stones as well as the tension against the adversary ones. Definition of energy function stands back algorithms to compute the power of stones patterns at the successive Go states, so account the each state dominance. A Go game phase-transition-like process happens when after a movement the black—white force equilibrium is broken and emerges pre-eminence of blacks over white or conversely [52].

The study of phase transitions in biological system can indicates fast changes in the metabolic or genomic landscape [53]. Previously related, the Ising model was used to model the cell pass from healthy to cancer as a phase transition [14]. As a general descriptor of interactions [54, 55], we use the Ising model as the approximation to describe the interaction of the cancer cells and healthy or immune cells and how some key event lead to the change from healthy to a metastatic cancer or its remission, so advantage its comprehension.

### Cancer versus immune system formalisms

Recent modelling of metastasis includes the use of game theory parameters [56], and differential or partial equations proposals to quantify the impact to the cells a given element known to be present in metastatic niches [57], or the conversion of healthy cells to the cancer phenotype [14]. These approaches are limited in the number of features they can use or using overcomplicated models. With the Ising model, we flexible can introduce several features related to the cancer metastasis process or to the immune system reaction behavior. The Go game outline a suggestive simulation of the interaction between two types of cells. The modeling of the Go game by Ising Hamiltonian gives the advantage of controlling the weight of the different features that are relevant to model interaction event by event. This type of control also allow further research concerning how big a cluster of black-cancer cells is at a given moment, and how it continues its growth or how it shrinks for the pressure of white-immune elements.

## Materials and methods

Now we describe the Ising Hamiltonian, first for modeling Go black versus white stones fighting, then for modeling the cancer process versus the immune system reaction.

### The energy function for Go gaming

The energy function in the Ising Hamiltonian uses the common fate graphs (CFG) representation of Go states [58]. CFG is an useful technique for grouping stones in Go as well as for establishing the neighborhood relationships among them during the game. It makes easy to deal with the interaction among allied stone, versus adversaries, or with liberties involved. Associated to Ising Hamiltonian in Eq (1) for modeling Go gaming the energy function embraces the next parameters:

- The numbers of atomic (single) stones in a molecular (compound) stone.
- The number of eyes a stone is involved to.
- The tactic pattern the stone is making.
- The strength of ally stones that have synergy among them.
- The strength of ally stones that counterbalance the synergy of adversary stones.

To get this goal, we propose the quantitative description of stone utilizing the elements involved in Eq (2):

$$\begin{array}{c} x_{i}=c_{i}(n_{i}+r_{eye}^{k_{i}}) \end{array}$$ (2)

*n*_*i*_ sets the number of single stones, *r*_*eye*_ is the constant to represent the occurrence of an eye, *r*_*eye*_ > 1, or *r*_*eye*_ = 0 if no eye; *k*_*i*_ sets the number of eyes in stone *i*, and *c*_*i*_ is the stone color, 1 for white, and −1 for black. Hence, $r_{eye}^{k_{i}}$ quantifies the impact of the number of eyes in *i*, and *k*_*i*_ ≥ 2 says that *i* could never be captured as having 2 not removal liberties. If no eye *x*_*i*_ just indicates the *i* size and color. In Hamiltonian of Ising model for Go parameter, *w*_*ij*_ sets the ratio of union or repulsion between each pair *i*, *j* of single or compound stones. So *w*_*ij*_ encompasses tensions alongside paths joining stone *i* to *j*, being affected by the presence and strength of adversary stones that may impede the *i* − *j* connection; or, on the opposite, by the presence of allied stones that result in mutual strengthen. Up to rules and tactics in Go gaming, the feature interaction among stones is assessed utilizing next Eq (3):

$$\begin{array}{c} w_{ij}=\sum_{s}r_{t}x_{s}^{ij} \end{array}$$ (3)

$x_{s}^{ij}$ describes each stone *s* lying between *i* and *j*, that makes a tactic pattern; *r*_*t*_ sets a quantify of a-priori known power of the pattern *t* eye (*r*_*eye*_), net (*r*_*net*_), ladder (*r*_*lad*_), invasion (*r*_*inv*_), reduction (*r*_*red*_). Pattern parameters fit a total order > induced by a-priori knowledge of Go tactics power, by an averaging procedure from real matches between top level players estimation open to analysis and precisions: we say that an eye tactic has top power, followed by a net, a ladder, an invasion and a reduction. Thus, *r*_*eye*_ > *r*_*net*_ > *r*_*lad*_ > *r*_*inv*_ > *r*_*red*_. Single liberty parameter value is *r*_*sl*_ = 1.

First term of Hamiltonian in Eq (1) accounts the interaction of collaboration among patterns of same color stones, or the fight against adversaries; for the second term, the particular external field *h*_*i*_ adds the number of liberties the stone *i* has. Henceforth, given any Go game state, by definitions in Eqs (2) and (3) used in Eq (1) we quantify the power of each color set of stone on the base of: the each stone size and, by quantifying, positive with allies and negative with adversaries, the interaction energy within the tactic the stones are entangled. So, the synergy with allies within each tactic of invasion, reduction, connection, eye, ladder or net pattern; and, the negative tension under the pressure of the adversary tactics. It assimilates the Ising-model-based algorithms to approach the growth, metastasis and control of cancer process.

### Cancer invasion, metastasis and the immunity reaction as Go tactics

**Fig 1:**
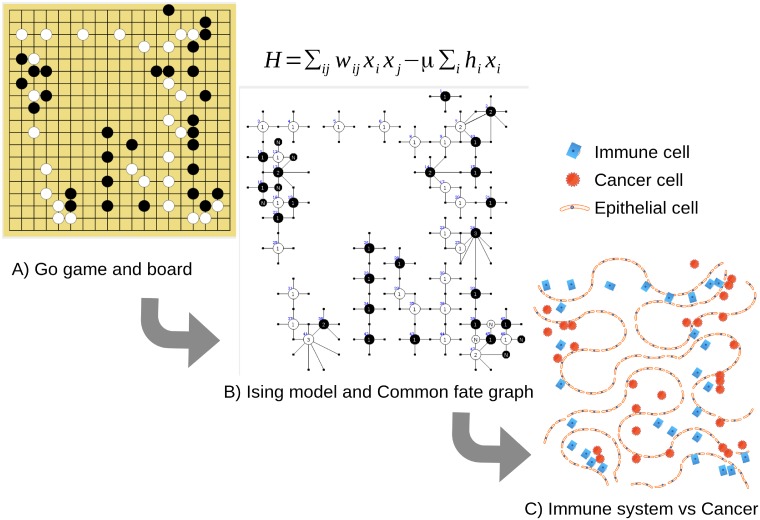
Scheme of cancer as Go gaming. Go gaming cancer processes: (A) Go game in an intermediate state having black—white dominance in equilibrium; (B) Ising hamiltonian and the CFG from the mentioned state; (C) over tissues, the cancer cells expansion versus the immune system behave.

For making the analogy between the cancer metastasis and Go gaming process, we observe that the Go board is extensive in size to treat it as a tissue or composite organization of epithelial cells, fibrin, and ECM. The black stones encase several types of cancer cells, like the CTCs, tumour-initiating cells, and the solid tumour cells. On the other side, the white stones encase several types of tumor suppressor barriers like the activation of PTEN [59], the *p*19^*ARF*^ pathway [60], natural killer cells, cytotoxic T cells, and treatments like chemo- or radiotherapy. Of course, in the Go game, both the black and white stones have the same type and number of strategies, and the weight of these strategies is also the same. In the interplay between the cancer cells and the immune system the number of strategies naturally vary, we assume those are the same number, but a relevant difference with the previous analysis of Go gaming is the weight that these strategies can have. The Go counterpart of the cancer processes and the proposed comparison follows. The Fig 1 illustrates the scheme of cancer as Go gaming: The dynamics of Go—cancer are CFG depicted and quantified by the Ising Hamiltonian, then we illustrate this cancer cell spreading and the fight with therapies or immune system.

Each stone is formally described in Eq (2), and up to if cancer (black) or immune (white) cells (stones) these are the parameters involved in. Eq (3) sets the formal account of interaction among cells. In Table 1 the cells and biochemical processes participating in the cancer growth are listed, each matching the respective Go tactic. In Table 2 the biochemical elements and processes participating in the immunity reaction are listed, each matching the respective Go tactic.

**Table 1:** Correspondence between cancer and immune system tactics to Go tactics.

Go tactic	Cells involved in cancer	Cancer tactic	Assigned value
Eye	CTCs	Initial micro tumours	0.7
Net	Tumour cells	Premetastatic niche—solid tumour	0.6
Ladder	Tumour cells, stromal cells	Tumour secreted factors and extracellular vesicles	0.5
Connection	Stromal cells vicinity	Metastatic microenvironment setup	0.1
Invasion	Tumour initiating cells	Primary invasion	0.4

**Table 2:** Correspondence between cancer and immune system tactics to Go tactics.

Go tactic	Cells involved in immune system	Immune system tactic	Assigned value
Eye	Anti-tumoural pathway signaling activation, macrophages and NK cells	tumour suppressor barriers	0.4
Net	Cytotoxic T cells	Cytotoxicity	0.7
Ladder	Macrophages and NK cells	A set of protected tissue from the cancer invasion	0.5
Connection	Helper T and B cells	Antigen presentation and cell signaling	0.1
Reduction	Chemo- or radio-therapy, T and B cells	Arrival of chemicals or immune system cells	0.5

To associate the Ising Hamiltonian in Eq (1) to cancer modeling, the energy function should embrace the next parameters:

1. *Invasion*: abnormal cells appear in the vicinity of the tissue or “sedding”.
2. *Reduction*: Immune cell system, chemo- or radio-therapy try to deactivate or disappear cancer cells in the proximity.
3. *Eye from black stones*: micro-tumours appear in the tissue.
4. *Eye from white stones*: tumour suppressors barriers appear in the tissue.
5. *Ladder from black stones*: cancer cells surrounded vicinity.
6. *Ladder from white stones*: immune system cells surrounded vicinity.
7. *Net*: a vicinity surrounded by cancer or immune cells in a loosen up manner.
8. *Connection*: between each other group of cancer or immune cells.
9. *Atari*: a cancer cell group adjacently-surrounded by healthy cells.
10. *Ko*: force equilibrium in a region of the tissue.

The cancer initial setup to win most of the area is analogous with the existence of the PMNs, these sites created by the action of primary tumors give an advantage to the invasion of CTCs [45]. This cancer metastasis process correlates some of the Go tactics into the next steps:

1. Tumor presence.
2. Neoangiogenesis.
3. PMN formation as a biochemical process.
4. Disequilibrium between MMP and TIMP as described in [56].

In the Go board right side in Fig 1(A) black stones make a strong dominance net in this zone, that hardly might be reduced by white stones. Since the cancer perspective, we could consider this zone as a (long) PMN thanks to a robust net by cancer cells, that easies the eventual growth of solid tumors. This net strategy, very characteristic in Go gaming for board area domination has a direct interpretation in the cancer process: it corresponds to a PMN that induces cancer metastasis. It is well known that cancer cells are good for invasion but weak for colonization: The PMN provides the conditions for metastasis success. The detailed interaction of immune system tactics versus cancer tactics is described by the next paired scenarios:

- Tumour cell seeding (invasion) engage in a confrontation with arrival immune system cells or therapies (reduction).
- A cancer tumour (net of cancer cells) engage in confrontation with cytotoxicity (net of the immune system and healthy cells).
- Tumour secreted factors, and extracellular vesicles (ladder of cancer factors) engage in confrontation with a set of protected tissue from the cancer invasion (ladder of immune system and healthy cells).
- Stromal (net of) cells vicinity engage in confrontation with antigen presentation and cell signaling (net of immune system)

### Ising Hamiltonian for cancer and for immune system

The quantitative description of cancer versus immune system phenomena via the elements involved in Eq (2) follows: *n*_*i*_ sets the number of single or compound cells, *c*_*i*_ indicates the group of the cell, 1 for the immune system, −1 for cancer. The presence of an eye *r*_*eye*_ is as explained before with *k*_*i*_ the number of eyes in the group *i*. Thus, $r_{eye}^{k_{i}}$ quantifies the impact of the micro-tumor in the tissue for cancer, or the elements strengths for the immune system reaction. The bigger the *k*_*i*_ the more strong the vicinity there for cancer or for the immune system.

In addition, in Eq (3), *w*_*ij*_ sets the ratio of synergy or repulsion of the interaction between each pair *i*, *j* of single or compound cells. Paths between cells of the same group make synergy. And the presence and strength of cells of the adversary group make tensions by the obstruction of the synergy between *i* − *j*: $x_{s}^{ij}$ describes each single or compound cells *s* that may interfering with (a tactic pattern) adversary. *r*_*t*_ is the assigned weighting from the strategy *t*: eye (*r*_*eye*_), net (*r*_*net*_), ladder (*r*_*lad*_), invasion (*r*_*inv*_), reduction (*r*_*red*_). We followed the hierarchy of Go strategy for the three top strategies *r*_*eye*_ > *r*_*net*_ > *r*_*lad*_. Concerning invasions and reductions in the context of cancer may carry a different weight. Whenever strength of reduction from the immune system has a relevant moment and advantage over the invasion due the effectiveness of the molecular mechanism activated to suppress tumors, *r*_*inv*_ < *r*_*red*_, or conversely. The connection between same kind of cells results in a new cells arrangement. So, connection effect is quantified from the size and tactic the new cells composition is making.

Henceforth, for modeling cancer versus immune system, the use of Eqs (2) and (3) in Eq (1) measures the interaction strength among all of the allied or adversary groups of cells. It is an expression of the contribution of each cancer or immune system tactic: likely cancer/immune system eye, ladder or net, as well as the invasion, reduction or connection of cancer tumors; or, the against coordinated reaction from the elements of the immune system. Eq (3) quantifies the synergy of cells from the same type, among the tumors or among the immune system elements, or the fight tension between adversaries.

### Scenarios and simulations

We used an in-house Ising model simulator, programmed to use four different behavior scenarios

1. Random versus Random; as a control of the system
2. Aggressive black player (GNUgo) versus passive White player (Montecarlo); Representing a dominance of cancer cells
3. Good White player (Smart) versus Aggressive black player(GNUgo); Representing a clear state of metastasis
4. Aggressive white player (GNUgo) versus Aggressive black player (GNUgo); Representing an equilibrium between strong cancer and a strong immune system

We performed several games using combinations between these four players. We evaluated all saved games using the Ising model presented previously. We displayed different scenarios according to the tactics from cancer or the immune system. The different scenarios responded to 3 different notions:

1. A strong player vs. a weak player that correspond representing metastatic cancer vs. the weaken immune system or vice-versa the control of cancer cells/tumours by an immune system/therapy,
2. Equal level players that correspond to a moment in which cancer and immune system is in a tight, stable relationship, and
3. A random chance simulation that corresponds to the control scenario.

100 games between the different players and saved each game in the.sgf format. The evaluation of the Ising model was performed by giving different weights to the tactics to reflect the importance of an event over the other with the values describes earlier n this section.

We made the energetic description of the events of the multiple invasions in our simulation with the Ising model. The winner of the simulation is the group (cancer cells or immune system) with most negative value for the Ising energy.

## Results

### The energy of a random system

**Fig 2:**
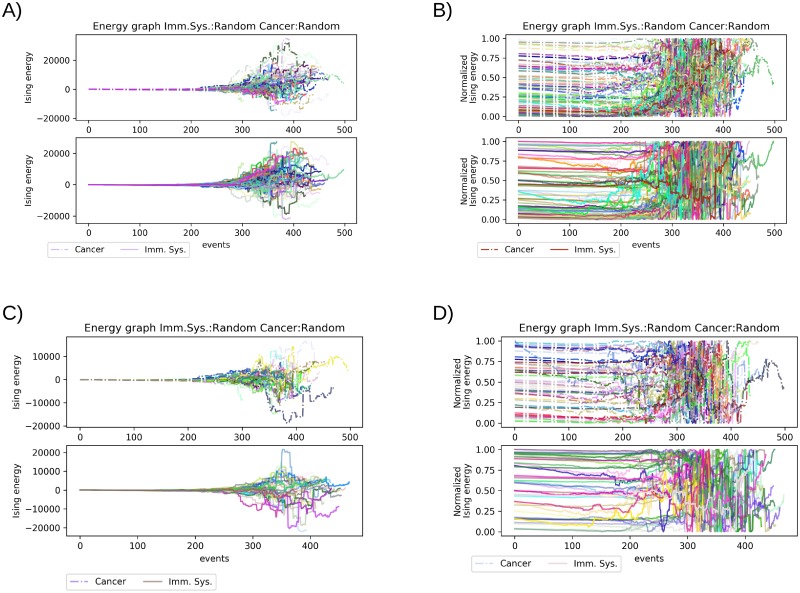
Comparison of the random vs. random scenario. A) A plot of the Ising energy of all the simulations put together. B) Normalized Ising Energy of all the simulations show in A. C) The upper panels show only the simulations won by the cancer cells and the lower panel show only the simulation won by the immune system. D) Normalized Ising energy of the simulation displayed in C in the same order upper panel corresponds to simulation won by the cancer cells, and the lower panel simulation won by the immune system.

The control scenario of random invasions/reductions showed that each of the simulations would find have phase transitions between the 200 and 300 events. Between the 300 and the 400 event several sudden steep energy changes happened in a very short span of time, the changes indicate that several phase transitions occur as a result of the irregular distribution of the stone/cells in the board. In the random scenario no simulation ended before the 300 events, in total 55 out of 100 of the simulations resulted in the cancer cell winning through random invasion, however, there is no trend in their energy landscape. The energy distribution reflects the movement on the board in a stochastic way, variating in the number of events it takes to end the simulation (Fig 2A). The normalized values of the Ising energy allows the contrast of each of the simulations, but more importantly, it let us locate the phase transition points in this kind of staked plot. Fig 2 presents a staked plot of all the simulation for the random versus random scenario. In http://dx.doi.org/10.17504/protocols.io.nqddds6 the individual resulting plots for the 100 simulations for each scenario are shown.

The Ising energy values of the cancer cells or the immune system are close to zero for the most of the simulation, and usually, this is a draw between the strategies of the two players. The draw indicates that the system maintains equilibrium approximately up to the 200 events. However, this long equilibrium in the random scenario is due to the lack of strategies (Fig 2B). Additionally, we extracted the simulations won by cancer cells or the immune system (Fig 2C and 2D). The idea behind the isolation of the corresponding simulation was to observe the energy landscape of the winners and features shared among both winners. With this separation, we can see that the most prominent positive value corresponds to one simulation in the immune system player, although some events later it rapidly decreases and won the match. This sudden change indicates that the immune system cells are in apparent disarray allowing the cancer cells (black player) to take advantage in this stage then turning the tables and winning the match. Many simulations reached high Ising energy values, but only a handful of the simulation reach Ising energy beyond 10 000 units (Fig 2C). Using the normalized values of the energy shows only some of the winner’s simulation present sharp Ising energy variations within the first 200 events (Fig 2D). Further, for most of the simulations, the changes start near to the 200th event, and the most prominent changes happened just before the 300th event, but still, no trend could be established from isolating the winners of these simulations.

Being a random scenario the Ising energy takes any value according to the state of the system. This show that the Ising model describes what is happening in the interplay of all cells in a random cancer cell seeding scenario. The simulations demonstrate that only seeding events do not have enough energy drive in all simulation to favor the cancer cells. Also, we can apply the same criteria for the immune system, where it shows that an uncoordinated immune system is unable to defend the invasion of cancer cells.

### Aggressive player as template for the metastasis or a strong immune system

**Fig 3:**
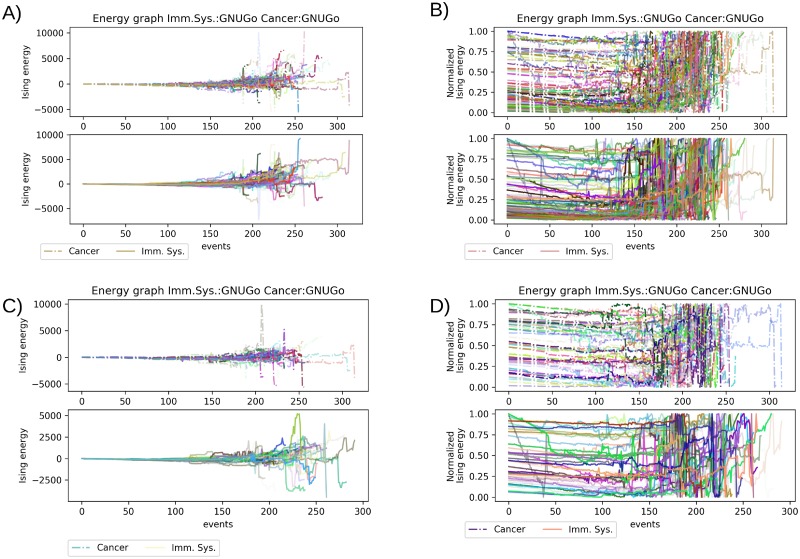
Comparison of the aggressive player vs passive player scenario. A) Plot of the Isign energy for all the simulations between GNUGo and MonteCarlo, upper panel correspond to cancer ells energy lower panel correspond to immune system energy. B) Normalized values of the simulations in the same order as A.

As expected the aggressive player won most of the simulations against a good or a passive player. First, we consider the scenario of the aggressive player versus the passive one (Fig 3). When the aggressive player was against the passive player, that is represented by a Monte-Carlo based simulator; we notice the trend where most occasions the aggressive player dominates the board before the 100 events (Fig 3A). We also notice that any of the simulations acquired greater Ising energy values than the random scenario. Using the normalized value of the Ising energy, we can see that after the 200th event that all the simulation have a steep rise in their values almost in an exponential shape (Fig 3B). The aggressive player won these simulations 98.5% of the time. We observed the same trend in the complementary scenario where the aggressive player represents the cancer cell invasion and metastasis. Here, in this scenario, the cancer cells won 97 of the simulation hence causing metastasis, and the immune system won only 3 of these simulations. In this scenario where few events quickly result in favor to the aggressive player, the energy landscape of the system always produces very high values. The energy values indicate that the aggressive player prepares the board in its favor, the presence of PMN for cancer cells or an immune system primed with anti-cancer vaccines.

### Aggressive and good player resemble the remission or spike in metastasis

**Fig 4:**
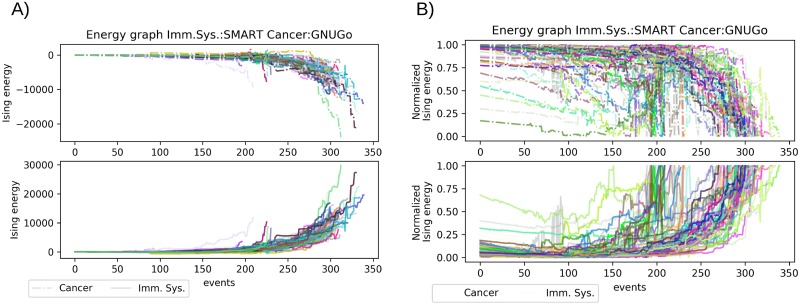
Comparison of the aggressive player vs. good player scenario. A) Plot of the Ising energy for all the simulations between GNUGo and SMART, upper panel corresponds to cancer cells energy lower panel correspond to immune system energy. B) Normalized values of the simulations in the same order as A.

In another scenario where we have a good player, represented by a SMART simulator, the aggressive player won 97% of all the simulations, and the Ising energy shows the good player holding a more extended stalemate with the aggressive player than the passive player but shorter than the random player. In general terms, the Ising energy of this scenario is close to the random scenario limits, except for one simulation that ends up with the highest and lowest value of all simulation. The Ising energy indicates that the draw between the aggressive and the good player is maintained close to the 150th event (Fig 4A). After the 200th event, the energy values of the simulation are separated favoring the aggressive player. However, in many of the simulations after the 200th event, the good player was able to hold the aggressive player into a stalemate. With the normalized values of Ising energy, we noted several abrupt changes in the energy in very short time span for the aggressive player, indicated in Fig 4B lower panel as the immune system. Comparing the upper and lower panel of Fig 4B shows the presence of two groups of simulation in the aggressive player. The first group starts with some advantage, and before the 150th event, there are several of these changes mention before, after this stage the simulation comes to an end. The second group of simulation is at equilibrium with their counterparts, in this group those changes in the energy happen after the 250th event and then there is an exponential decrease in Ising energy. On the other hand, in the upper panel the passive player displays a similar energy landscape to the passive player. We noted that for the good player there is an inflection point for most of the simulation between the 100 and 150 events, after this part, it appears to be an exponential rise in the normalized Ising energy.

### Energetic landscape of the aggressive player as immune system and metastatic cancer

**Fig 5:**
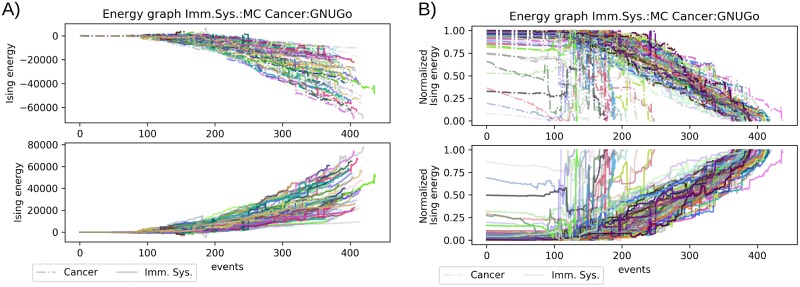
Comparison of the aggressive player vs aggressive player scenario.

So far, we used the aggressive player acting only as cancer cell spreading or as the response of the immune system. Now, we used the aggressive player to represent both categories in the same match. In these scenarios which portrait a strong immune system fighting off an aggressive cancer metastasis, the energetic description of the simulations showed a behavior different to the random seeding (Fig 5). In total cancer cells won 55 out of the 100 simulations and the immune system 44 out the 100. In this scenario we normalized the Ising energy (Fig 5B and 5D) and again, isolated the simulation won by each player (Fig 5C and 5D). Again, the major differences in the energy landscape started after the 100th event, until that point the immune system and the cancer cells were in a stalemate similar to the scenario of the passive player. Regardless the winner of the simulation, due to the change in the number of cancer or immune cells, there are several shifts in the overall energy of the system leading to lower values of energy compared with the other scenarios (Fig 5A and 5C). These shifts are mostly present after the 150th event and appear even until the end of the simulation. This fluctuation in the energy makes difficult predict a clear winner during the simulation. The normalized value shows that between the 150th and 250th event there is a big number of rapid changes in the Ising energy landscape. These big number of changes indicates the importance of the initial setup in the first 100-150 events since after this number of events the final competition for space depends on the positions of the allied cells (Fig 5B). The sudden changes in energy can be better appreciated in the normalized energy values of the isolated winners (Fig 5D). Here, like in the random scenario, there is no trend in the energy landscape, the only apparent difference is that between the 150-250 events the simulations won by the immune system are sparser than the simulation won by cancer. That indicates that cancer did not have a better initial setup than the immune system, forcing a close interchange in the energetic advantage with its opponent, the immune system.

A comparative summary about the time events spend by the cancer damage versus the immune system capacity of reaction is in Table 3. The strong the cancer the speed it uses to growth and damage, but should be regarded, as well, the strength of the immune system to react to. The both elements trade-off determines the time events spent in each of the simulated scenarios.

**Table 3:** This table shows the percentage of simulations ended in before a given number of events. The label meaning is as follows: Strong corresponds to the GnuGo simulator, Medium correspond to the SMART simulator and the Weak correspond to the MonteCarlo simulator. IS correspond to immune system and C correspond to Cancer.

Time simulation	100	150	200	250	300	350	400	450	500
Random IS vs random C:	0	0	0	0	2	0	60	0	38
Weak IS vs aggressive C:	0	0	15	0	6	0	54	0	25
Medium IS vs aggressive C:	0	0	7	0	59	0	34	0	0
Strong IS vs aggressive C:	0	0	18	0	80	0	2	0	0
Strong IS vs weak C:	0	0	0	0	7	0	21	0	72
Strong IS vs medium C:	0	0	3	0	65	0	32	0	0

## Discussion

We tangle with the idea that some events in cancer are similar to plays in Go game. Spreading of cancer starts by invading another space quite far from the point of origin, that is metastasis. Similarly, in Go the black stones’ player, usually, fix next stones very far from previously placed. The Ising model allows us to describe the phenomena of the interplay of cancer cells and immune system using a theoretical approach on how such binary system behave regarding an energy landscape. In Physics, forces in conflict, in quasistationary equilibrium, are frequently associated with processes in the critical zone where qualitative transitions take place. Perhaps, we might consider critical zone dynamics in further complex diseases modelling.

We think each simulation as a patient our model allows us to observe the key events that derive in a remission or worsening of the patient at the end. The use of Ising model allows giving attention to the difference between scenarios where the cancer is so aggressive that -in our simulations- it takes less than 150 (time) events to take control of the board, therefore, winning the simulation. The Ising energy landscape of the random scenario can be compared to the scenario when the aggressive player is both the immune system and the cancer cells. In both scenarios we were unable to find a trend in the Ising, there is one common characteristic: during about 100 events there is a substantial exchange of energy between the immune system and the cancer cells. However, the aggressive player has lower limits of the Ising energy and shorter span of time (number of events).

In contrast, the other two scenarios with a good and a passive player have similar characteristics. In both scenarios the Ising energy landscape adopts large values, especially in the passive player scenario, adding to that both show a trend in the energy landscape of an exponential increase of the Ising energy after the 100-150th events.

Also, we can make the analogy that the case of a more passive immune system, one that is not primed or boosted to fight cancer, we observed it takes a shorter time (number of events) for aggressive cancer to gain on the board area making metastasis and winning the match/simulation. This characteristic might be well a reflex of the initial conditions set by the aggressive cancer nursing a beneficial environment for the next invasion. Here the energies of the simulation won by aggressive cancer takes considerable negative values, indicating that the preceding steps give a distinct advantage in the system to cancer spreading. Still, the very few simulations won by the passive player acting as the immune system takes positive values, ending the simulation with a value near to zero. This difference tells us there are particular configurations of the spreading of cancer that allows openings to change its zone of influence and can be neutralized by the immune system, modifying the energy landscape from a very disadvantageous to something more favorable in a very short span of time.

One of the most interesting scenarios is the aggressive player versus itself taking the role of cancer and immune system. This scenario is analogous to the cancer is aggressive enough in a person with a strong immune system. It is already mentioned that the Ising energy has many sharp changes in short periods of time or number of events, this in can be interpreted as the emergence of mutations in the cancer cells or the boost of the immune system.

While we have used the concept of seeding, the exact number of seeding events for aggressive cancer cannot be determined experimentally. However, studies in vitro of metastatic melanoma showed the appearance of probable melanoma cell colonies that later cannot be found in the tissue after some days possibility invading tissue underneath [61]. In fact, our simulations are in 2D and resemble a lot the spreading of the cancer cell in a monolayer tissue, the introduction of the new dimension to make the model 3D would enhance our simulation by allowing us to study the vertical invasion of cancer cells. In the metastatic process, we might have metastasis in more than one place. In such a case, one might need to model no a binary process but an n-ary one with n greater or equal to 3. There exists a generalization of the Ising model, the Potts model [62] where a general number, n, of states is considered. Ising model corresponds to n equal to 2. Including the 3D component in further modelling is a significant step to refine our description of the cancer metastasis.

The current clinical treatments for cancer have a low efficacy rate because the patient’s body is treated as a whole, and the chemo- or radio-therapy negatively affects, both the tumor and the healthy tissues. A reasonable clinical alternative is that the patient receive selective therapy for the cancer tissues and not the whole body are being proposed [38, 63, 64]. Most of the approaches explore this possibility with the mathematical models and computer simulations to find what is called intelligent therapies. In [65] a differential equation modeling for chemotherapy include the parameters of: the growth rate of the tumor, the cycle-specific chemotherapy, the fraction of cells that are (not) in a cell-cycle phase affected by the chemotherapy, the drug sensitivity of the cells, and the drug concentration in the tumor; as well as the set of differential equation for the EBRT (external beam radiation therapy) includes logarithmic and exponential functions for growth and reduction of tumors. However, like most approaches, the model is for a single primary tumor without considering the specific location of the tumor and leaving the treatment of metastases in some specified area of an organ out of consideration. The modeling of cancer is complex and the available information on therapies treatment is scarce or with high variance. The inclusion of these therapies for the modeling of cancer metastasis is a major constraint in the current analysis for the phenomena. Prediction of metastasis is a central challenge to overcome. We propose with our abstraction a way to depict the growth and localization of several tumors besides the primary invasion. The metaphor of an organ as part of the board in the game can help in the future to model a site specific therapeutic treatment. All the parameters in the [65] model can be included in an ad-hoc Ising Hamiltonian function definition.

## Conclusion

Go game and the Ising model provide the elements to advance the characterization of cancer invasion, reduction and metastasis in various scenarios. This hybrid approach, focused in the interaction of the tumor cells versus the healthy tissues, has the flexibility to add the diverse elements participating in the cancer process and in the reaction of the immune system to. So, it goes beyond from the use of differential or partial equations. Using the Ising model, the updates in the energy function for cancer, the progression of the invasion following is explicit. As well, it gives insight into different scenarios where cancer can spread in less than 100 events, overwhelming the immune system of a person. Furthermore, we lay out scenarios where the immune system can hold the cancer progression at the same speed or numbers of events in the simulation. Next work should include statistical analysis with a step by step description of the events, as well to increase the number of the parameters used in the Ising model with a smaller system. This manner we could explore the minimum number of events that end up with the aggressive player winning, and expand the modelling from 2D to 3D.
